# Assessment of the Postpartum Emotional Wellbeing among Women Participating and Not Participating in Antenatal Classes

**DOI:** 10.3390/ijerph19084476

**Published:** 2022-04-08

**Authors:** Anna Kucab, Edyta Barnaś, Joanna Błajda

**Affiliations:** Institute of Health Sciences, Collegium Medicum, University of Rzeszow, 35-959 Rzeszow, Poland; ebarnas@interia.eu (E.B.); jblajda@ur.edu.pl (J.B.)

**Keywords:** pregnant, antenatal class, Edinburgh Postnatal Depression Scale (EPDS)

## Abstract

The perinatal period is associated with an increased risk of emotional disorders. Exposure to stress impacts the functioning of the maternal brain, also shaping the developmental processes of the child’s brain. To assess the emotional wellbeing of women participating and not participating in antenatal classes. The study involved a group of 200 women divided into two groups: the study group, participants of the antenatal classes (N = 100), and the control group, not participating in the antenatal classes (N = 100). The Edinburgh Postnatal Depression Scale (EPDS) and Mini-COPE inventory were used. EPDS was administered at two time points: on the 2nd day of puerperium and 6 weeks after the delivery, while Mini-COPE inventory was applied once during pregnancy. Severe symptoms of depression on the 2nd day after childbirth concerned 16.0% of the women (N = 15) participating in the antenatal classes and 11.0% of the controls (N = 11). Intensification of depressive symptoms 6 weeks after the delivery occurred in 19.5% of the females attending antenatal classes (N = 17) and 18.8% of the controls (N = 18). Severe symptoms of depression 6 weeks after the delivery in the group of women participating in antenatal classes were significantly related to adopting helplessness to cope with stress and a sense of being accepted by the husband/partner, family, and society. In the case of women not participating in antenatal classes, the severity of symptoms of depression 6 weeks after the delivery was slightly related to the sense of acceptance by the husband/partner, family, and society. Apart from identifying risk factors for emotional disorders in pregnancy, it is worth taking into account whether a woman participated in antenatal classes when planning the care in the perinatal period. This factor can be a potential protective factor in preventing emotional problems after childbirth.

## 1. Introduction

Pregnancy is a complex and sensitive period that presents many challenges for women, including the development of postpartum mental disorders (PPDs). These conditions may range from postpartum depression and anxiety, which are relatively common, to less frequent but more severe postpartum psychosis [1,2].

Perinatal depression is common worldwide, with an overall prevalence of 11.9% of women, and this percentage was even higher during the 2019 coronavirus outbreak [3]. Since it is associated with the leading cause of disability and burden of disease worldwide, perinatal depression poses an important public health problem [4]. Intense maternal sadness and anxiety may prevent her from bonding with her baby or breastfeeding. In severe cases, women may think about hurting themselves or their baby. Untreated maternal depression may result in low birth weight and impairment of the child’s social, cognitive, and emotional development [5].

Concomitant anxiety and perinatal depression require clinical attention given the potential negative developmental consequences for the baby. More research is needed to develop evidence-based interventions to prevent, identify, and treat this comorbid disease [6,7].

Screening for the symptoms of perinatal depression during pregnancy enables healthcare professionals to implement preventive and clinical measures. The risk of perinatal depression is higher in pregnant women with more severe depression and anxiety symptoms [8].

Patients with perinatal depression may demonstrate some or many of the following symptoms: sadness, crying, depressed mood, tearfulness, anxiety, indifference, insomnia or excessive sleep, lack of attachment, or interest in the child [5].

These mental symptoms may pose a serious obstacle in fulfilling the role of a mother, as they hinder the emotional relationship with the child, affect the quality of its care, and lower the self-esteem of women and their faith in a successful close relationship with the child. Susceptibility to parental stress is an important predictor of the quality of the parent–child relationship. The shaping of the maternal attitude implies, among many sociodemographic, personality, and psychological factors, also the psychological well-being of a woman already during pregnancy [9].

According to a South African study, maternal depression not diagnosed or treated early resulted in negative consequences for children, such as malnutrition and mental, social, and physical health and even death. The long-term effects of untreated maternal depression include impaired cognitive development, language acquisition and deviant behavior, and unfavorable economic conditions later in life [10].

Personal resources are undoubtedly one of the important factors influencing the effectiveness of coping with stress. They allow us to recognize a situation as stressful or not, influencing strategies undertaken and responses [11].

Due to serious consequences, it is important to take preventive measures aimed at early detection and treatment when the first symptoms appear.

The process of mothers and fathers’ preparation for childbirth and parenthood should be individualized, systematic antenatal education conducted by appropriate specialists and based on real interaction [12].

Contemporary prenatal education in the form of antenatal classes is aimed at the theoretical and practical preparation of women and their partners for the best possible parenting. Antenatal classes have been a key part of maternity care for many years and are designed to help to prepare women and their partners for childbirth. The results identified as important include: gaining knowledge about childbirth, birth method, and self-efficacy in relation to childbirth, which may have a secondary impact on the woman’s birth experience [13].

In this study, an attempt was made to assess postpartum emotional wellbeing of women using the Edinburgh Postnatal Depression Scale (EPDS) at two time points postpartum as a screening tool to assess the risk of depression symptoms and the Mini-COPE inventory used once during pregnancy to measure one-time coping with stress. Additionally, the respondents were divided into participating and nonparticipating in antenatal classes.

## 2. Purpose of the Study

The aim of the study was to assess the emotional wellbeing of women after childbirth who participated or did not participate in antenatal classes.

### Research Hypotheses

**Hypothesis 1 (H1).**  *Participation in antenatal classes has a positive impact on the emotional functioning of pregnant and postpartum women*.

**Hypothesis 2 (H2).**  *The presence of symptoms indicating emotional disorders during pregnancy has an influence on the severity of these symptoms after childbirth*.

## 3. Materials and Methods

Ultimately, the cohort study included 200 women, residents of the city and the Rzeszów powiat, who took part in all three stages of the study. The respondents were a representative group for the population of women giving birth in four hospitals in the city of Rzeszów. It was established that in 2016, there were 9128 deliveries in hospitals in Rzeszów (with a confidence interval of 0.95% and a maximum error of 0.05). The study was conducted from October 2016 to December 2018. The women were divided into two equal groups: the study group, participants of the antenatal classes (N = 100), and the control group, women not participating in the antenatal classes (N = 100).

The study was designed in three stages.

Stage 1—concerned pregnant women. Mini-COPE inventory was administered.

Stage 2—women on the 2ndday postpartum were asked to complete EPDS.

Stage 3—women in late puerperium (6 weeks postpartum) were asked to complete EPDS.

The study was approved through a resolution from the Bioethics Committee of the University of Rzeszów on 8 June 2016 (5 June 2016). After being informed about the course and purpose of the study, the respondents expressed their informed consent in writing to participate in the study. The inclusion criteria for the study were: informed consent to participate in the study, adult women, logical verbal contact, pregnant women, and puerperium—participants of antenatal classes and not participating in antenatal classes (enrollment took place through the Obstetrics and Gynecology Clinic, Department of Pregnancy Pathology, Provincial Clinical Hospital No. 2, in Rzeszów). The classes covered the prenatal period (pregnancy), childbirth, postpartum period (puerperium), and social support for the pregnant woman, obstetrician, and the family in the perinatal period. The basic topics of the antenatal school included issues related to the development of the child in the womb, the period of pregnancy, the course of childbirth and the role of the father in childbirth, the physiology of puerperium, breastfeeding and latching on to the breast, care and nursing of the newborn, and bathing the newborn. The antenatal school also takes into account the aspect of returning to a psychophysical wellbeing after childbirth. Women in the second trimester of a physiological pregnancy are admitted to antenatal classes, after presenting a certificate from an obstetrician-gynecologist regarding attending gymnastics. Antenatal schools usually offer group and individual classes. The meetings are usual held twice a month and hosted by a specialist (midwife, doctor, physiotherapist, lactation consultants, psychologist).

The method used in the study was a diagnostic survey, and the techniques—the Edinburgh Postnatal Depression Scale (EPDS) and the Mini-COPE inventory—for measuring coping with stress.

EPDS is used as a screening test for maternal symptoms of postpartum depression. It was developed in Livingston and Edinburgh in 1987 by Cox et al. The scale consists of 10 short items; a mother responds to every question by herself by choosing one of four possible answers that best describes her feelings over the past 7 days [14]. Mothers with a borderline score (12, 13 points out of 30 possible) are likely to suffer from depressive disorders of varying severity. In doubtful cases, the test is repeated after 2 weeks. EPDS is specifically designed to detect postpartum mood disorders and cannot be used to detect anxiety or personality disorders in a young mother.

The tool has good psychometric properties. A validated Polish version of EPDS is available. In the original studies, the sensitivity amounted to 86%, the specificity was 78%, and the Cronbach’s alpha reliability coefficient was 0.88 [15].

The Mini-COPE inventory is intended mainly for research purposes, although it can also be used in practice in screening tests. It is a tool for examining adults—both healthy and sick. It consists of 28 statements grouped in into 14 strategies (2 statements in each strategy). This method is most often used to measure one-time coping, that is, to assess typical ways of reacting and feeling in situations of severe stress. The emerging differences in how women cope are likely to depend on the difficulties they experience [11].

The assumptions of homogeneity of variance were checked by Levene’s test, For the 2nd stage for EPDS Cronbach’s alpha = 0.869, for the 3rd stage for EPDS Cronbach’s alpha = 0.903. The normality of distributions by the Kolmogorov–Smirnov test.

### Statistical Analysis

The results were presented using the methods of descriptive statistics: mean, standard deviation, median, minimum and maximum value, and 95% confidence interval for the mean. The significance level in the analysis was set at *p* < 0.05. The analysis was based on IBM SPSS Statistics 20 using the Pearson’s chi-squared test of independence, McNemar test, and linear regression using the stepwise method. Initially, statistical analysis was performed using the input method. Factors important for EPDS were identified sequentially, and then only those that actually influenced EPDS were taken into account, the most important of which were distinguished by stepwise analysis.

## 4. Results

The study group consisted of 200 women, half of whom were participants of the antenatal classes. The average age of women participating in antenatal classes (29.87 ± 4.40 years) was slightly higher than the average age of women not participating in these classes (28.57 ± 5.61 years). Significant differences were noted in the case of the place of residence—the antenatal classes were more often attended by female residents of large cities (N = 45, i.e., 45.0%), while women from rural areas participated in them less frequently (N = 62, i.e., 62.0%). These classes were also attended more often by women with higher education (N = 78, i.e., 78.0%) as well as those employed and having a partner (N = 81, i.e., 81.0%). Significant differences also concerned the mean duration of the relationship, it was longer in the group of women who did not participate in antenatal classes (6.28 ± 4.25 years) than in the respondents who participated in these classes (5.26 ± 3.38 years) (Table 1).

For women who participated in antenatal classes, the current pregnancy was more often planned (N = 85, i.e., 85.0%) than for women not participating in these classes (N = 69, i.e., 69.0%). Additionally, a positive attitude towards the present pregnancy was presented more often by women participating in antenatal classes (N = 90, i.e., 90.0%) compared with those who did not participate in these classes (N = 78, i.e., 78.0%). The course of pregnancy was not significantly (*p* = 0.4105) related to participation in antenatal classes (Table 1).

Subsequently, it was verified how the selected variables influence the severity of depression symptoms on the 2nd day of the puerperium and 6 weeks after the delivery among women participating and not participating in antenatal classes. It was shown that in the group of women participating in antenatal classes, the influence of variables on the severity of depression symptoms on the 2nd day of the puerperium was not noticeable. In the case of women who did not participate in antenatal classes, it was noticed that the severity of depression symptoms on day 2 after the puerperium was significantly related to feelings/emotions in the first days after delivery (*p* = 0.0014)—women who negatively assessed their feelings after childbirth had a greater severity of depression symptoms before delivery. The severity of depression symptoms 6 weeks after the delivery in the group of women participating in antenatal classes was significantly associated with the choice of helplessness coping strategy (*p* = 0.0324) and the sense of acceptance by the husband/partner, family, and society (*p* = 0.0047). Women participating in antenatal classes experienced greater severity of depression symptoms as they coped with difficult situations through helplessness and felt less accepted by their husband/partner, family, and society. In the case of women not participating in antenatal classes, the severity of depression symptoms 6 weeks after the delivery was slightly associated with a sense of acceptance (*p* = 0.0448)—higher severity of depression symptoms correlated with a lower sense of acceptance by the husband/partner, family, and society (Table 2).

The severity of depression symptoms on the 2nd day of the puerperium concerned 16.0% of women (N = 15) participating in antenatal classes and 11.0% of the respondents (N = 11) who did not participate in these classes. Intensification of depression symptoms 6 weeks after the delivery occurred in 19.5% of females attending antenatal classes (N = 17) and 18.8% of females (N = 18) not participating in antenatal school. The mentioned differences were not statistically significant (*p* > 0.05) (Table 2).

The severity of depression symptoms on the 2nd day of the puerperium among women participating in antenatal classes was higher when their living conditions were worse (*p* = 0.0129), pregnancy had a negative impact on their professional career (*p* = 0.0393), and the sense of coherence was decreased (*p* = 0.0010). In the group of women not participating in antenatal classes, the higher level of depression severity on the 2nd day of the puerperium was associated with the accumulation of negative feelings in the first days after delivery (*p* = 0.0001), more frequent coping with difficult situations through helplessness (*p* = 0.0052), and to a lesser extent, active coping (*p* = 0.0306).

The severity of symptoms of depression in 6 weeks after the delivery in the group of women participating in antenatal classes increased with the lack of a sense of acceptance by the husband/partner, family, and society (*p* = 0.0012) and coping with helplessness (*p* = 0.0117), and it decreased with an increasing sense of coherence (*p* = 0.0216) and coping with a sense of humor (*p* = 0.0461).

In the case of female respondents who did not participate in antenatal classes, the severity of depression symptoms 6 weeks after the delivery was greater when women experienced the negative impact of pregnancy on partnerships more (*p* = 0.0161), more often used the strategy referred to as helplessness (*p* = 0.0132), less frequently coped actively (*p* = 0.0402) (Table 3).

## 5. Discussion

The aim of this cohort study was to assess the emotional wellbeing of women in the perinatal period using the EPDS screening questionnaire and to assess the ways of coping with stressful situations using the Mini-COPE inventory. The study included women attending antenatal classes and women not using this form of education. It is worth noting that antenatal classes were held in a traditional way, in direct contact, so the participants could initiate a dialogue with the teachers, freely ask questions, and be among other parents.

The program, which includes classes at the antenatal school, is designed not only to prepare parents to care for a newborn and infant. Moreover, it helps them to deal with negative emotions: it tames the fear of childbirth and teaches how to deal with pain and excessive and unnecessary fear and how to achieve a sense of harmony and peace in the experience of childbirth.

Shorey et al. investigated the effectiveness of a technology-based supportive parenting education program (SEEP) on parental outcomes at four time points—during pregnancy (third trimester), 2 days postpartum, 1 month postpartum, and 3 months postpartum—and demonstrated that SEEP is effective in improving parental bond, perceived social support, and parental satisfaction and reducing postpartum depression and postpartum anxiety [16].

The transition to parenting can be stressful for new parents. Poor parental adjustment can, in turn, lead to negative parental outcomes and adversely affect a child’s development. Easily accessible technology-based educational programsare needed to support parents in the crucial period of the perinatal period, enhancing the future well-being of the family and facilitating a smoother transition to parenting [16]. In Poland, women after childbirth are guaranteed continuity of care through a patronage visit by a community midwife. It is free and available to all women.

The present study showed that more than half of the women who participated in antenatal classes mentioned the reduction of anxiety associated with childbirth/cesarean section (N = 68, i.e., 68.0%) among the benefits of these classes (N = 68, i.e., 68.0%). Similar results were obtained by Pinar et al. and Karabulut et al., who found that participants of antenatal classes had less fear of childbirth and were characterized by a higher level of knowledge and faster adaptation to pregnancy and the postpartum process [17,18]. According to Lowdermilk et al., antenatal education plays an important role in preparing participants for pregnancy, childbirth, and parenthood [19]. Information obtained during childbirth classes reduced anxiety during pregnancy and childbirth among women surveyed by Miquelutti et al. [20]. According to Antonovsky and Sagy, antenatal education equips participants with information resources and practical coping skills [21]. Byrne et al. and Toohill et al. demonstrated a positive effect of antenatal education on the reduction of fear of childbirth [22,23].

In Poland, a new organizational standard of perinatal care has been in force since 2019, which includes recommendations for screening tests using EPDS twice during pregnancy and once after childbirth, without indicating the day/week after childbirth when it should be performed (Regulation of the Minister of Health of 16 August 2018 on the organizational standard of perinatal care. Journal of Laws 2018, item 1756) [24]. The current study project was completed 6 months before the effective standard came into force. It is also worth paying attention to recent reports that clearly define that the early part of the puerperium is not suitable for screening based on EPDS.

The use of the Edinburgh Postnatal Depression Scale (EPDS), originally designed for outpatient screening for postpartum depression (PPD), does not predict the development of PPD when the questionnaire is used during hospitalization shortly after delivery. According to a study by Ezirim et al. EPDS results obtained 3 to 24 h postpartum did not reliably predict the elevated results 6 weeks later [25].

In a study by Tariq et al., women were tested twice on the Edinburgh 10-Point Postpartum Depression Scale (EPDS): in the third trimester of pregnancy and then 2 weeks after delivery, when they returned for a follow-up visit [26]. Additionally, Srkalović Ismiragić et al. used EPDS within 3–5 days after delivery and 6–9 weeks after delivery [27].

The differences occurring in our study are the result of the project implementation before the publication of the results of the above-mentioned authors and before the entry into force of the Polish organizational standard of perinatal care.

It is worth emphasizing that the use of the EPDS scale was and is intended to be of a screening nature only, indicating the identification of a risk group that should be subject to specialist consultation.

The most frequently mentioned risk factors for the development of perinatal depression are: family history, socioeconomic factors, and the patient’s attitude towards the current pregnancy. It is difficult to compare the results of our study when comparing the respondents participating and not participating in antenatal education with the literature, because the available reports concern respondents without such a division.

In a study by Fiala et al. in univariate and multivariate analysis, risk factors for the development of postpartum depression (PPD) were identified, and it was shown that the main factors of PPD are: previous depressive episodes, experiencing psychosocial stressors by women, dissatisfaction with being pregnant, and lack of a life partner. As the risk of depression is associated with the experience of psychosocial stressors, it seems important to encourage women at risk to use counseling in antenatal classes [28]. A study of women in Pakistan found a significant association of PPD with low socioeconomic status and with unplanned pregnancies. In addition, antenatal depression was strongly associated with postnatal depression, indicating that the former is a significant predictor of the latter [26]. Similar data were obtained from a systematic review from India and Japan where international differences in psychosocial factors of perinatal depression were analyzed. It has been shown that the common factors associated with the onset of perinatal depression for women from both countries were are demographic factors, such as young age of the mother, financial problems, unwanted pregnancy, and family history of mental problems [29].

A primiparous woman may show general anxiety about her new situation, and in her next pregnancy, she may experience all sorts of emotions resulting from the experiences of previous births. This study correlated the relationship between the number of pregnancies and the level of depression after childbirth. Fewer pregnancies corresponded to a higher level of postpartum depression (*p* = 0.0131). In a study by Ayele et al., one of the factors increasing the likelihood of depression was the woman’s first pregnancy, which could be associated with various psychosocial problems, as well as fear of complications during pregnancy [30].

A similar result was obtained by Satoh et al. pointing to a relationship between the birth of the first child and the occurrence of postpartum emotional disorders [31]. The results of a longitudinal study of women in the third trimester of pregnancy show a higher probability (*p* < 0.05) of perinatal depression in women whose pregnancy was another unplanned one, or they belonged to middle and lower socioeconomic classes [26].

In a study by Guo et al. carried out in China, no increased risk of perinatal depression was observed in women giving birth for the second time compared with women giving birth for the first time; moreover, the EPDS results among women giving birth for the second time decreased in the perinatal period [32]. This is an extremely interesting report, taking into account the context of the sociopolitical situation in this country.

Preferred coping styles in difficult situations were assessed on the basis of the Mini-COPE inventory. Coping methods such as active coping, planning, or seeking instrumental support are treated as problem-focused strategies. On the other hand, strategies such as seeking emotional support, turning to religion, and denial are among the assumed emotion-oriented strategies. On the other hand, discharge, dealing with something else, cessation of activities, taking psychoactive substances, and sense of humor are considered less effective, although in some situations very useful [11].

The choice of coping strategies in difficult situations among the surveyed women did not significantly depend on their participation in antenatal classes. In both groups, a higher level of depression symptoms correlated with the choice of helplessness strategy, which may be expressed by the lack of adaptation to a difficult situation. It is believed that active strategies are associated with less stress, while as the levels of stress increase and the disease gets worse, avoidance strategies begin to dominate [33].

Social support has a slightly different aspect influencing coping with stress not only in women in the perinatal period. This is confirmed by the results of a study by Satoh et al., which aimed to assess the postnatal experiences of Asian mothers at risk of postpartum depression (PND) and the insights of peer volunteers about the technology-based peer support intervention program (PIP). Mothers who received PIP had less negative feeling sand a better understanding and acceptability of one’s emotional situations; this allowed them to enjoy their birth experiences better. Mothers without additional support felt lost, anxious, and lonely [16].

Social support significantly affects the quality of coping with stress in pregnant women, increases the chances of its successful completion, and minimizes fears and negative emotions. The source of support for women is primarily their immediate environment: partner/spouse, family, friends, midwife, and doctor. Social support from a partner/spouse is rated higher than that obtained from other people, as it has a positive effect on the emotional state of a pregnant woman. It minimizes the intensity of stress, anxiety, and depression, which in turn is associated with the successful completion of labor [34]. This is an important aspect in the context of both parents’ participation in antenatal education, as it strengthens the bond between parents and indirectly influences parenting.

## 6. Limitations

The study was conducted only in one center; however, it was a third-level reference hospital, which is representative of the Podkarpacie region, Poland. Research should be extended to include other centers, both in Poland and abroad.

The Edinburgh Postnatal Depression Scale Questionnaire was applied on the 2nd day and the 6th week after childbirth. According to the latest studies, EPDS results obtained within month after delivery are not reliable due to the high frequency of transient mental disorders.

An additional limitation is the lack of measurement of the emotional state with EPDS during pregnancy. It is worth noting that the study originated and was conducted in the period before the implementation of the organizational standard of perinatal care in Poland (2019), which assumes a triple assessment of the emotional state of a woman.

## 7. Conclusions

1. Participants of the antenatal classes more often presented a positive attitude towards the present pregnancy.
2. Women not participating in the antenatal classes, who negatively assessed their feelings after childbirth, demonstrated higher severity of depression symptoms before childbirth.
3. The severity of postpartum depression symptoms increased in both groups when women more often declared a sense of helplessness in difficult situations and less frequently coped actively.

## Figures and Tables

**Table 1:** Characteristics of the respondents with a breakdown into the group participating in antenatal classes (study group) and the group not participating in antenatal classes (control group).

		Groups				*p*
		Study Group		Control Group		
		N	%	N	%	
Age (mean ± SD; min.–max.)	29.22 ± 5.07; 17–41	29.87 ± 4.40; 21–39		28.57 ± 5.61; 17–41		0.0698
Age	Up to 25 years	13	13.0	26	26.0	0.1182
	26–30 years	47	47.0	40	40.0	
	31–35 years	30	30.0	23	23.0	
	Over 35 years	10	10.0	11	11.0	
Place of residence	Town	19	19.0	22	22.0	<0.0001
	City	45	45.0	16	16.0	
	Rural area	36	36.0	62	62.0	
Education	Primary	0	0.0	1	1.0	0.0140
	Vocational	2	2.0	5	5.0	
	Secondary	20	20.0	37	37.0	
	Higher	78	78.0	57	57.0	
Living conditions	Good	89	89.0	85	85.0	0.4003
	Average	11	11.0	15	15.0	
	Bad	0	0.0	0	0.0	
	Financial problems	0	0.0	0	0.0	
Source of income	Both partners employed	81	81.0	59	59.0	0.0023
	One partner employed	18	18.0	36	36.0	
	Other	1	1.0	5	5.0	
Type of relationship	Marriage	89	89.0	84	84.0	0.1910
	Partnership	11	11.0	13	13.0	
	n/a	0	0.0	3	3.0	
Duration of the relationship (mean ± SD; min.–max.)	5.76 ± 3.86; 0.25–20	5.26 ± 3.38; 1–15		6.28 ± 4.25; 0.25–20		0.0627
Planned pregnancy	Yes	85	85	69	69	0.0072
	No	15	15	31	31	
Attitude of the woman to the present pregnancy	Positive	90	90.0	78	78	0.0206
	Negative	10	10.0	22	22	
Course of the present pregnancy	Uneventful pregnancy	42	42.0	41	41.0	0.4105
	Increased-risk pregnancy	49	49.0	44	44.0	
	High-risk pregnancy	9	9.0	15	15.0	

**Table 2:** The severity of depressive symptoms (EPDS) among women participating and not participating in antenatal classes (linear regression using the introduction).

**EPDS**			**2nd Day of the Puerperium**				**6 Weeks after the Delivery**			
**Antenatal Classes**			**Yes**		**No**		**Yes**		**No**	
**Adjusted Rate**			**Beta**	***p***	**Beta**	***p***	**Beta**	***p***	**Beta**	***p***
(Constant)				0.8934		0.3619		0.5793		0.3718
Active coping			−0.021	0.8719	−0.233	0.0871	0.036	0.7422	−0.169	0.2354
Helplessness			0.176	0.2363	0.176	0.2385	0.265	0.0324	0.241	0.1274
Sense of humor			−0.166	0.2120	0.039	0.7689	−0.189	0.0854	−0.185	0.1914
Sense of coherence			−0.251	0.1100	0.069	0.6201	−0.205	0.1121	−0.074	0.6131
Age			0.070	0.5790	0.124	0.3256	−0.137	0.1900	0.004	0.9767
Place of residence			0.037	0.7748	0.086	0.5144	−0.137	0.2029	−0.105	0.4483
Education			−0.101	0.4650	0.131	0.3276	0.093	0.4124	−0.141	0.3196
Living conditions			0.224	0.1264	0.039	0.7504	0.107	0.3692	−0.124	0.3345
Sense of acceptance on the part of the husband/partner, family, society			−0.052	0.7471	−0.042	0.7333	0.388	0.0047	0.268	0.0448
Feelings/emotions in the first days after childbirth			0.060	0.6778	0.397	0.0014	0.106	0.3745	−0.013	0.9182
Dealing with existing problems			0.169	0.2301	0.136	0.2694	0.010	0.9312	0.204	0.1183
Partnership relations			0.138	0.3490	0.055	0.6398	0.069	0.5654	0.209	0.0987
Life plans			−0.106	0.4830	−0.130	0.2868	−0.035	0.7739	−0.190	0.1397
Professional Carter			0.213	0.1201	0.154	0.2260	0.082	0.4631	0.065	0.6288
Family relations			0.118	0.4595	0.083	0.4889	0.053	0.6835	−0.065	0.6074
			0.454		0.454		0.624		0.395	
**R^2^**			**2nd Day of the Puerperium—Scale Results**				**6 Weeks after the Delivery—Scale Results**			
			**Yes**	**No**	***p***		**Yes**	**No**	***p***	
Participation in antenatal classes	Study group	N	15	79	0.3111		17	70	0.8920	
		%	16	84			19.5	80.5		
	Control group	N	11	89			18	78		
		%	11	89			18.8	81.3		

**Table 3:** Severity of depression symptoms among women participating and not participating in antenatal classes (linear regression using the stepwise method *).

**2nd Day of the Puerperium**			**Nonstandardized Coefficients**		**Standardized Coefficients**	**T**	***p***
			**B**	**SE**	**Beta**		
Groups	Study group (R^2^ = 0.348)	(Constant)	14.607	6.094		2.397	0.0199
		Sense of coherence	−0.115	0.033	−0.393	−3.477	0.0010
		Living conditions	5.605	2.183	0.292	2.568	0.0129
		Professional career	2.488	1.179	0.230	2.110	0.0393
	Control group (R^2^ = 0.376)	(Constant)	1.664	3.326		0.500	0.6186
		Feelings/emotions in the first days after childbirth	7.460	1.770	0.424	4.214	0.0001
		Helplessness	3.335	1.152	0.300	2.894	0.0052
		Active coping	−2.441	1.103	−0.230	−2.213	0.0306
**6 Weeks after the Delivery**			**Nonstandardized Coefficients**		**Standardized Coefficients**	**T**	***p***
			**B**	**SE**	**Beta**		
Groups	Study group (R^2^ = 0.516)	(Constant)	15.918	6.448		2.469	0.0166
		Sense of coherence	−0.092	0.039	−0.271	−2.364	0.0216
		Sense of acceptance on the part of the husband/partner, family, society	3.315	0.970	0.349	3.418	0.0012
		Helplessness	3.915	1.503	0.278	2.606	0.0117
		Sense of humor	−2.015	0.988	−0.198	−2.040	0.0461
	Control group (R^2^ = 0.260)	(Constant)	−1.082	5.334		−0.203	0.8399
		Helplessness	3.494	1.370	0.290	2.550	0.0132
		Partnership relations	11.250	4.549	0.272	2.473	0.0161
		Active coping	−2.728	1.302	−0.237	−2.095	0.0402

* Factors important for EPDS were identified sequentially, and then only those that actually influenced the EPDS were taken into account, the most important of which were distinguished by stepwise analysis.

## Data Availability

The authors can be contacted for information on the dataset.

## References

[1] Meltzer-Brody S., Howard L.M., Bergink V., Vigod S., Jones I., Munk-Olsen T., Honikman S., Milgrom J. (2018). Postpartum psychiatric disorders. Nat. Rev. Dis. Primers.

[2] Dye C., Lenz K.M., Leuner B. (2022). Immune System Alterations and Postpartum Mental Illness: Evidence From Basic and Clinical Research. Front. Glob. Womens Health.

[3] Hu M., Zhou Y., Xue M., Ren Y., Li S., Wang R., Qi L., Zeng L., Liu Z., Qian W. (2022). The prevalence and correlates of peripartum depression in different stages of pregnancy during COVID-19 pandemic in China. BMC Pregnancy Childbirth.

[4] Camoni L., Mirabella F., La Sala L., Romano G., Barbano G., Cattaneo M., Cena L., Michielin P., Ialenti V., Veltro F. (2021). Feasibility and effectiveness of the Australian perinatal mental health approach in the Italian health services: Progress and challenges. Ann. Ist. Super Sanita.

[5] Van Niel M.S., Payne J.L. (2020). Perinatal depression: A review. Cleve Clin. J. Med..

[6] Falah-Hassani K., Shiri R., Dennis C.L. (2017). The prevalence of antenatal and postnatal co-morbid anxiety and depression: A meta-analysis. Psychol. Med..

[7] Dadi A.F., Miller E.R., Mwanri L. (2020). Postnatal depression and its association with adverse infant health outcomes in low- and middle-income countries: A systematic review and meta-analysis. BMC Pregnancy Childbirth.

[8] Mikšić Š., Miškulin M., Juranić B., Rakošec Ž., Včev A., Degmečić D. (2018). Depression and Suicidality during Pregnancy. Psychiatr. Danub..

[9] Teti D.M., Towe-Goodman N., Gonzalez M. (2019). Postpartum Depression, Effects on Infant. Reference Module in Neuroscience and Biobehavioral Psychology.

[10] Mokwena K.E. (2021). Neglecting Maternal Depression Compromises Child Health and Development Outcomes, and Violates Children’s Rights in South Africa. Children.

[11] Juczyński Z., Ogińska-Bulik N. (2012). Tools for Measuring Stress and Coping with Stress.

[12] Haapio S., Kaunonen M., Arffman M., Åstedt-Kurki P. (2017). Effects of extended childbirth education by midwives on the childbirth fear of first-time mothers: An RCT. Scand. J. Caring Sci..

[13] National Guideline Alliance (UK) (2021). Antenatal Classes: Antenatal Care: Evidence Review E.

[14] Cox J.L., Holden J.M., Sagovsky R. (1987). Detection of postnatal depression. Development of the 10-item Edinburgh Postnatal Depression Scale. Br. J. Psychiatry.

[15] Hewitt C.E., Gilbody S.M., Mann R., Brealey S. (2010). Instruments to identify post-natal depression: Which methods have been the most extensively validated, in what setting and in which language?. Int. J. Psychiatry Clin. Pract..

[16] Shorey S., Ng Y.P.M., Ng E.D., Siew A.L., Mörelius E., Yoong J., Gandhi M. (2019). Effectiveness of a Technology-Based Supportive Educational Parenting Program on Parental Outcomes (Part 1): Randomized Controlled Trial. J. Med. Internet Res..

[17] Pinar G., Avsar F., Aslantekin F. (2018). Evaluation of the Impact of Childbirth Education Classes in Turkey on Adaptation to Pregnancy Process, Concerns About Birth, Rate of Vaginal Birth, and Adaptation to Maternity: A Case-Control Study. Clin. Nurs. Res..

[18] Karabulut Ö., CoşkunerPotur D., DoğanMerih Y., CebeciMutlu S., Demirci N. (2016). Does antenatal education reduce fear of childbirth?. Int. Nurs. Rev..

[19] Lowdermilk D.L., Perry S.E., Cashion K., Alden K.R. (2012). Maternity& Women’s Health Care.

[20] Miquelutti M.A., Cecatti J.G., Makuch M.Y. (2013). Antenatal education and the birthing experience of Brazilian women: A qualitative study. BMC Pregnancy Childbirth.

[21] Antonovsky H., Sagy S. (1986). The development of a sense of coherence and its impact on responses to stress situations. J. Soc. Psychol..

[22] Byrne J., Hauck Y., Fisher C., Bayes S., Schutze R. (2014). Effectiveness of a Mindfulness-Based Childbirth Education pilot study on maternal self-efficacy and fear of childbirth. J. Midwifery Women’s Health.

[23] Toohill J., Fenwick J., Gamble J., Creedy D.K., Buist A., Turkstra E., Ryding E.L. (2014). A randomized controlled trial of a psycho-education intervention by midwives in reducing childbirth fear in pregnant women. Birth.

[24] Regulation of the Minister of Health, 16th August, 2018 on the Organizational Standard of Perinatal Care and Stands since 1st January 2019. https://isap.sejm.gov.pl/isap.nsf/download.xsp/WDU20180001756/O/D20181756.pdf.

[25] Ezirim N., Younes L.K., Barrett J.H., Kauffman R.P., Macleay K.J., Newton S.T., Tullar P. (2021). Reproducibility of the Edinburgh Postnatal Depression Scale during the Postpartum Period. Am. J. Perinatol..

[26] Tariq N., Naeem H., Tariq A., Naseem S. (2021). Maternal depression and its correlates: A longitudinal study. J. Pak. Med. Assoc..

[27] SrkalovićImširagić A., Begić D., Šimičević L., Bajić Ž. (2017). Prediction of posttraumatic stress disorder symptomatology after childbirth—A Croatian longitudinal study. Women Birth.

[28] Fiala A., Švancara J., Klánová J., Kašpárek T. (2017). Sociodemographic and delivery risk factors for developing postpartum depression in a sample of 3233 mothers from the Czech ELSPAC study. BMC Psychiatry.

[29] Takegata M., Ohashi Y., Lazarus A., Kitamura T. (2017). Cross-National Differences in Psychosocial Factors of Perinatal Depression: A Systematic Review of India and Japan. Healthcare.

[30] Ayele T.A., Azale T., Alemu K., Abdissa Z., Mulat H., Fekadu A. (2016). Prevalence and Associated Factors of Antenatal Depression among Women Attending Antenatal Care Service at Gondar University Hospital, Northwest Ethiopia. PLoS ONE.

[31] Satoh A., Kitamiya C., Kudoh H., Watanabe M., Menzawa K., Sasaki H. (2009). Factors associated with late post-partum depression in Japan. Jpn. J. Nurs. Sci..

[32] Guo X.J., Chen J., Ren J.H., Deng X., Xu L.Z. (2020). Comparisons on perinatal depression between the first-child women and the second-child women in West China under the universal 2-child policy: A STROBE compliant prospective cohort study. Medicine.

[33] Olff M., Brosschot J.F., Godaert G. (1993). Coping styles and health. Personal. Individ. Differ..

[34] Sapkota S., Kobayashi T., Takase M. (2013). Impact on perceived postnatal support, maternal anxiety and symptoms of depression in new mothers in Nepal when their husbands provide continuous support during labour. Midwifery.

